# Results from a first-in-human phase I safety trial to evaluate the use of a vascularized pericranial/temporoparietal fascial flap to line the resection cavity following resection of newly diagnosed glioblastoma

**DOI:** 10.1007/s11060-024-04647-w

**Published:** 2024-04-26

**Authors:** Omer Doron, Tamika Wong, Faina Ablyazova, Souvik Singha, Julianna Cavallaro, Netanel Ben-Shalom, Randy S. D’Amico, Manju Harshan, Amy McKeown, Avraham Zlochower, David J. Langer, John A. Boockvar

**Affiliations:** 1 Department of Neurosurgery, Lenox Hill Hospital, Donald and Barbara Zucker School of Medicine at Hofstra/Northwell Health, 130 East 77Th Street New York,, New York, NY 10075 USA; 2 Department of Biomedical Engineering, The Aldar and Iby Fleischman Faculty of Engineering, Tel Aviv University, Tel Aviv, Israel; 3 Department of Pathology, Lenox Hill Hospital, Donald and Barbara Zucker School of Medicine at Hofstra/Northwell Health, 130 East 77Th Street New York,, New York, NY 10075 USA; 4 Department of Radiology, Lenox Hill Hospital, Donald and Barbara Zucker School of Medicine at Hofstra/Northwell Health, 130 East 77Th Street New York,, New York, NY 10075 USA

**Keywords:** Blood Brain Barrier, Glioblastoma, Temporoparietal Fascial Flap, Peri-Cranial Flap, Clinical trial

## Abstract

**Purpose:**

The efficacy of systemic therapies for glioblastoma (GBM) remains limited due to the constraints of systemic toxicity and blood–brain barrier (BBB) permeability. Temporoparietal fascial flaps (TPFFs) and vascularized peri cranial flaps (PCF) are not restricted by the blood–brain barrier (BBB), as they derive their vascular supply from branches of the external carotid artery. Transposition of a vascularized TPFF or PCF along a GBM resection cavity may bring autologous tissue not restricted by the BBB in close vicinity to the tumor bed microenvironment, permit ingrowth of vascular channels fed by the external circulation, and offer a mechanism of bypassing the BBB. In addition, circulating immune cells in the vascularized flap may have better access to tumor-associated antigens (TAA) within the tumor microenvironment. We conducted a first-in-human Phase I trial assessing the safety of lining the resection cavity with autologous TPFF/PCF of newly diagnosed patients with GBM.

**Methods:**

12 patients underwent safe, maximal surgical resection of newly diagnosed GBMs, followed by lining of the resection cavity with a pedicled, autologous TPFF or PCF. Safety was assessed by monitoring adverse events. Secondary analysis of efficacy was examined as the proportion of patients experiencing progression-free disease (PFS) as indicated by response assessment in neuro-oncology (RANO) criteria and overall survival (OS). The study was powered to determine whether a Phase II study was warranted based on these early results. For this analysis, subjects who were alive and had not progressed as of the date of the last follow-up were considered censored and all living patients who were alive as of the date of last follow-up were considered censored for overall survival. For simplicity, we assumed that a 70% PFS rate at 6 months would be considered an encouraging response and would make an argument for further investigation of the procedure.

**Results:**

Median age of included patients was 57 years (range 46–69 years). All patients were Isocitrate dehydrogenase (IDH) wildtype. Average tumor volume was 56.6 cm^3^ (range 14–145 cm^3^). Resection was qualified as gross total resection (GTR) of all of the enhancing diseases in all patients. Grade III or above adverse events were encountered in 3 patients. No Grade IV or V serious adverse events occurred in the immediate post-operative period including seizure, infection, stroke, or tumor growing along the flap. Disease progression at the site of the original tumor was identified in only 4 (33%) patients (median 23 months, range 8–25 months), 3 of whom underwent re-operation. Histopathological analyses of those implanted flaps and tumor bed biopsy at repeat surgery demonstrated robust immune infiltrates within the transplanted flap. Importantly, no patient demonstrated evidence of tumor infiltration into the implanted flap. At the time of this manuscript preparation, only 4/12 (33%) of patients have died. Based on the statistical considerations above and including all 12 patients 10/12 (83.3%) had 6-month PFS. The median PFS was 9.10 months, and the OS was 17.6 months. 4/12 (33%) of patients have been alive for more than two years and our longest surviving patient currently is alive at 60 months.

**Conclusions:**

This pilot study suggests that insertion of pedicled autologous TPFF/PCF along a GBM resection cavity is safe and feasible. Based on the encouraging response rate in 6-month PFS and OS, larger phase II studies are warranted to assess and reproduce safety, feasibility, and efficacy.

**Trial registration number and date of registration for prospectively registered trials:**

ClinicalTrials.gov ID NCT03630289, dated: 08/02/2018.

**Supplementary Information:**

The online version contains supplementary material available at 10.1007/s11060-024-04647-w.

## Introduction

Glioblastoma (GBM), the most common malignant primary brain tumor, is uniformly fatal despite conventional therapy with surgery, radiation, and chemotherapy. One of the difficulties in treating GBM stems from the intrinsic privileged nature of the brain, specifically as a result of the blood–brain barrier (BBB) [1]. This complex structure limits the passage of ionized, hydrophilic, and large molecules and limits systemic delivery of potentially effective chemotherapies from penetrating tumor tissue in vivo [2].

Several techniques have been investigated to overcome the BBB and facilitate drug delivery. These include the use of fusion proteins, viral vectors, convection-enhanced delivery, carrier-mediated transport systems, focused ultrasound, and intra-arterial delivery of osmotic agents [3–6]. However, these techniques have inherent shortcomings related to the delivery system, the drug itself, or its bioactivity. An ability to provide long-term durable delivery without requiring a specific drug or technique would permit more treatment versatility while allowing further clinical study comparing a variety of currently limited therapeutics.

Pedicled soft tissue flaps are commonly and frequently used in head and neck surgery and neurosurgery, reinforcing various skull base defects to prevent cerebrospinal fluid leaks [7]. Periosteal flaps and temporoparietal fascial flaps (TPFFs) are widely used options because they have predictable vasculature and a wide rotational arc [7, 8]. In cerebrovascular neurosurgery, flaps may be derived from regions of the superficial temporal artery (STA), internal maxillary artery, or omentum, rotated either directly or indirectly, and transposed onto ischemic brain regions [9–11]. A new vascular network develops between the flap and the brain tissue as neovascularization takes over. Following stroke or TBI, this neovascularization has been shown to lack many of the characteristics of the BBB and therefore creates a uniquely permeable vascular network within an otherwise privileged microenvironment [12].

We hypothesize that implantation of vascularized flaps into and lining the surgical resection cavity may promote similar neovascularization with the ability to deliver classically restricted chemotherapeutic agents and give tumor bed access to circulating immune cells without the constraints of the BBB, thus generating an opportunity to increase both the antitumoral immunogenic response as well as drug delivery into the GBM microenvironment. We, therefore, performed this first-in-human, phase I study to assess the safety and feasibility of placing a TPFF/PCF in the resection cavity of newly diagnosed GBM.

## Methods

### Patient eligibility

This study was approved by the Institutional Review Board (IRB) at the Feinstein Institute for Medical Research of Northwell Health. All patients provided written informed consent before entering into the study. Patients were recruited between November 2018 and November 2022. Adult patients (age > 18) expected to undergo a planned resection of known or suspected GBM were included if ≥ 80% resection of the enhancing region was considered safe and feasible pre-operatively. Additional inclusion criteria included a Karnofsky performance score (KPS) > 70 and a life expectancy greater than 6 months. Intraoperative histopathological analysis was required suggestive of WHO Grade IV glioblastoma (GBM) along with the operator’s assessment that a TPFF and/or PCF was technically feasible. Subjects were enrolled if they met the inclusion/exclusion criteria and agreed to participate by informed consent.

### Treatment plan & surgical technique

At baseline screening visits, patients were subjected to complete neurological and physical examinations as well as magnetic resonance (MR) imaging of the brain with contrast.

**Fig. 1:**
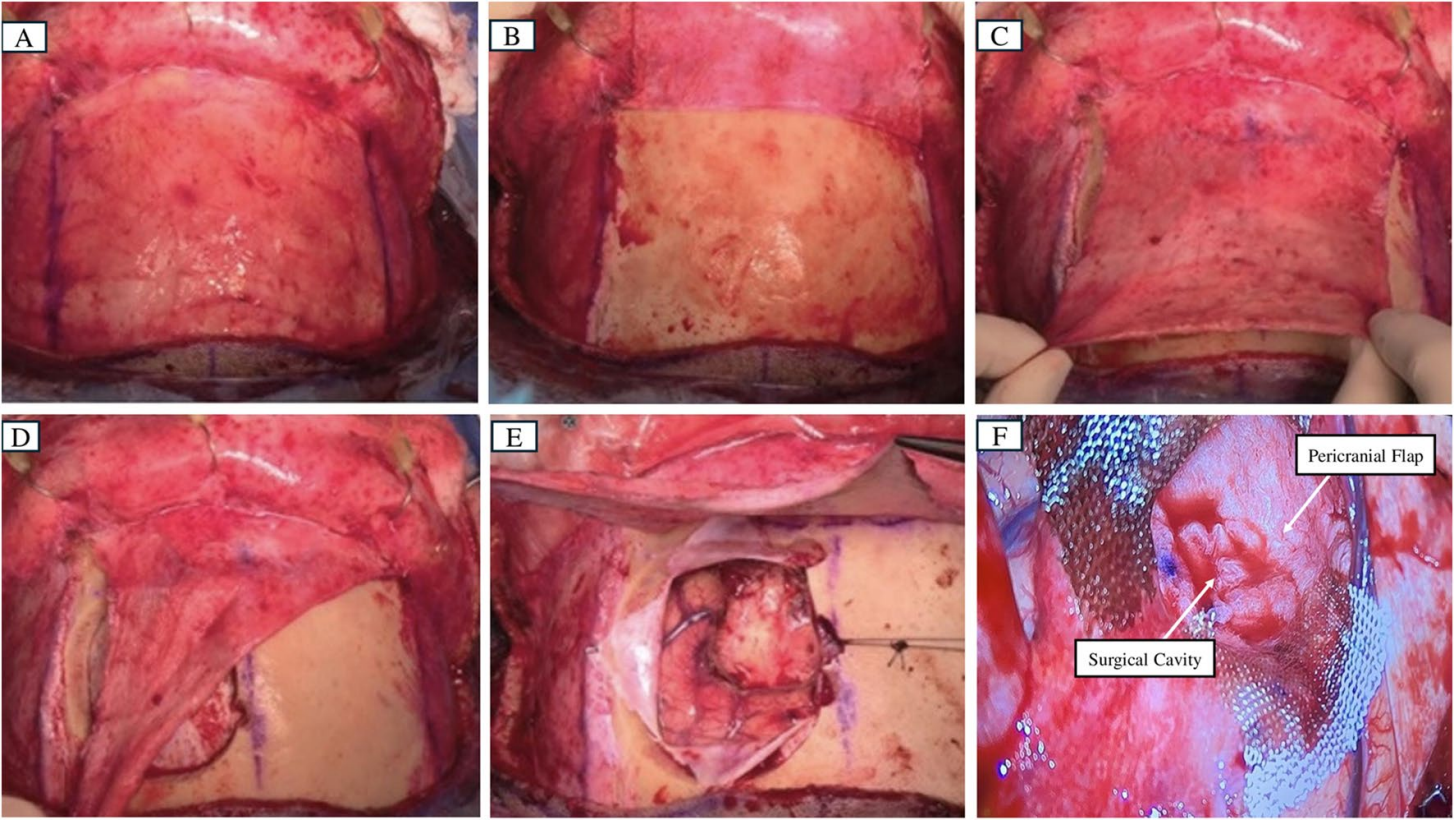
**A**, Pericranial flap was marked after making a bicoronal skin incision. **B**, Pericranial flap was harvested and reflected, exposing the craniotomy site. **C**, Pericranial flap was mobilized and tested for proper coverage. **D**, Pericranial flap was rotated towards the resection cavity. **E**, Exposed surgical cavity that would be lined by the flap **F**, Surgical cavity was lined by the pericranial flap

Day 0 was defined as the day of surgery. All subjects included in the study underwent standard safe, maximal resection of their brain tumor. All included patients had a histopathological diagnosis confirmed intraoperatively of GBM. Following resection, the surgical cavity was lined with a pedicled, autologous temporoparietal fascial flap or peri cranial flap (see Fig. 1). The patient's dura, bone, and scalp were closed in a customary manner sequentially with care not to put pressure on the pedicle of the flap. Details on the surgical implantation of the flap have been published previously [8].

11 patients underwent standard Stupp protocol with Temozolomide and radiation, 1 patient underwent hypofractionated radiation (4005 cGy in 15 fractions), and Temozolomide with clinical and radiological follow-up per clinical standard of care. Safety assessments were performed in the immediate post-operative setting and thereafter periodically, and consisted of a clinical and radiological evaluation of adverse events, tumor progression, and patient survival.

Safety was determined by assessment of the proportion of patients experiencing an increase in the frequency of seizures (defined as 15% relative to baseline), occurrence of a stroke, or occurrence of a severe infection, throughout the study duration.

Radiological progression was determined by the RANO (Response Assessment in Neuro-oncology) criteria utilizing the immediate postop MRI scan as the baseline study [13]. MRIs were acquired within 72 h post-op, at 30 days, 60 days, 90 days, and 180 days. Progression-free and overall survival (PFS and OS, respectively) from flap implantation functioned as a secondary outcome measure for this study.

### Statistical analysis

Progression-free survival was defined from the date of the surgery until the first documentation of disease progression, or death from any cause, whichever occurred first. Participants without documented disease progression at the time of analysis were censored at the time of their last follow-up. Overall Survival was defined from the date of surgery until death from any cause. All patients who were alive as of the date of the last follow-up were considered censored for overall survival. All statistical analyses were performed in SAS 9.4 (SAS Institute Inc., Cary, NC).

The study’s primary goal was to determine if a larger-scale trial was warranted based on these early results. For statistical calculation, we assumed that the median progression-free survival (PFS) for a patient newly diagnosed with glioblastoma (WHO IV) undergoing radiotherapy and concurrent chemotherapy with temozolomide is approximately 5–6 months, with 53.9% of patients having progression-free survival at 6 months [14].

With the proposed procedure of TPF flap implantation, we anticipated a 25% improvement in PFS, i.e. we believed that the median PFS would improve to 7–8 months and that approximately 65–75% of patients would be progression-free at 6 months. For simplicity, we then assumed that a 70% progression-free survival (response) rate at six months (midpoint of the anticipated 65% to 75%) would be considered a “favorable” or “encouraging” response to the new procedure.

Therefore, the criteria for moving ahead and conducting further studies on the TPF flap implantation would be if the response rate of this study was 70% or greater. Using a total sample size of ten subjects, observation of four or more responses would yield an exact 95% binomial which contains a value of 70%. While this result would not prove that the response rate is greater than 70%, it did, however, allow us to conclude that 70% was a plausible value, in which case we could make an argument for further investigation of the proposed TPF flap.

Thus, if we observed progression-free survival at six months in at least four subjects out of the first 10 participants, then this would provide sufficient evidence to warrant further investigation of the proposed TPF flap implantation. A subsequent investigational study such as a phase II trial or a randomized clinical trial could potentially be justified if we observed 4 or more subjects being progression-free at six months out of the total 10 participants. (Note that the four or more progression-free participants at 6 months need not be consecutive patients.)

## Results

### Patient demographics

The target enrollment for the study was 10 patients. 17 patients were screened, and 12 patients were enrolled. Screen failures included 5 patients, who were: patient 1 had a previous resection and the flap was not amenable for implantation; Patient 2: the frozen section could not confirm the presence of high-grade glioma; Patient 3: the implantation was not done because of the risk of implantation of the flap in an eloquent area; Patient 4: Frozen section was consistent with metastasis; Patient 5: tumor was left along the course of the middle cerebral artery (MCA), the decision was made not to place the flap over compromised MCA. Among the 12 patients in whom the flaps were implanted, 1 patient got a post-operative short course of radiation and 1 patient declined standard-of-care chemotherapy and radiation. So a total of 10 patients in whom pericranial flaps were implanted got standard of care Chemotherapy and radiotherapy. The clinical characteristics of all enrolled patients are presented in Table 1. Gross total resection of enhancing disease was achieved in all 12 patients and all had an uneventful post-operative course. Final histopathological analyses indicated IDH wildtype GBM (WHO Grade IV) in all patients. Most lesions were above 50 cm^3^ and presented with a mass effect on the initial MRI. The mean follow-up was 23.2 months (8.5 – 60.7 months). The final date of data collection and analysis was February 17, 2024.

**Table 1:** Patient and Tumor Characteristics

Age (years)	57 (46–69)
Gender (Male: Female)	9:3
Pre-Operative KPS^*^	90 (80–100)
Mean Follow-up (months)	23.2 (8.5 – 60.7)
Seizures at presentation	5/12
MGMT ( +)	5/10
IDH 1(-)	12/12
EGFR ( +)	7/10
Average lesion volume (cm^3^)	56.5

KPS: Karnofsky Performance Status

### Procedure and surgical approach

Tumor and treatment characteristics are shown in Table 2. Tumors were primarily located in the fronto-temporal regions. All the enrolled patients meeting the inclusion/exclusion criteria and agreeing to participate by informed consent were included in the study. The decision to use either the TPFF or PCF was at the discretion of the operating surgeon and depended mostly on the planned surgical approach. The pericranium is a direct extension and the distal end of a temporoparietal flap. Only if the lesion was at the frontal, vertex, or parasagittal regions of the skull, was the pericranial flap chosen due to its ability to reach those areas. TPPF was used in 5/12 cases, the rest utilizing a PCF.

**Table 2:** Tumor and Treatment characteristics pre- and post-operatively

Case No	Tumor Location	Lesion Volume (cm^3^)	Mass effect (Y/N)	Surgical Approach	Extent of Resection (%)	EBL (ml)	Follow up (months)	30-day Post operative complications	Length of Stay (Post operative day)	Functional status at discharge (KPS)	MRI at-3 months (RANO-based)	MRI at-6 months (RANO-based)	Infection & wound complications (Y/N)	Seizure control	Reoccurrence in craniotomy site (Y/N)	PFS (Months)	OS (Months)
1	Right Fronto-temporal	32	Y	Right Frontotemporal	100	200	17.2	None	5	80	Stable disease	Progression	N	Controlled with AED	N	3	17
2	Left temporo-occipital	26	Y	Left Posterior Temporal	100	150	60.7	None	8	80	Stable disease	Stable disease	N	Controlled with AED	Y	34	52
3	Right temporal	61	Y	Right Frontotemporal	100	110	13.1	None	5	100	Stable disease	Stable disease	N	Controlled with AED	N	8	12
4	Left Temporal	26	Y	Left Orbitotomy & Middle Fossa	100	200	33.6	None	8	70	Stable disease	Stable disease	N	Controlled with AED	N	33	33
5	Right Parietal	23	Y	Right Parietal	100	50	34.6	None	7	90	Stable disease	Partial Response	N	Controlled at an increased AED dose	Y	18	34
6	Left Frontal	44	Y	Left Frontal	100	100	13.0	None	6	80	Stable disease	Stable disease	N	Controlled with AED	Y	8	12
7	Right Parietal	132	Y	Right Parietal	100	50	25.9	None	3	80	Stable disease	Stable disease	N	Controlled with AED	N	9	17
8	Left Frontal	33	Y	Left Frontal	100	150	23.1	None	5	100	Stable disease	Stable disease	stitch abscess	Controlled with AED	N	12	14
9	Right Temporal	42	N	Right Temporal	100	300	18.5	None	18	80	Stable disease	Stable disease	N	Controlled with AED	N	9	9
10	Left Frontal	20	N	Left Frontal	100	100	15.8	None	5	80	Stable Disease	Stable Disease	N	Controlled with AED	N	8	8
11	Right Frontal	60	Y	Right Frontal	100	300	8.5	None	3	100	Stable disease	Progression	Pseudomeningocele	Controlled with AED	Y	7	7
12	Left Temporal	8	N	Left Temporal	100	150	14.8	None	6	80	Progression	Progression	N	Controlled with AED	Y	1	6

EBL: Estimated Blood Loss; KPS: Karnofsky Performance Status; RANO: Response Assessment in Neuro-Oncology; Y: Yes; N: No; AED: Antiepileptics drugs; PFS: Progression Free Survival; OS: Overall Survival

### Post operative period

No patient suffered from a new permanent neurological deficit following surgery during the hospitalization period. Functional status at discharge was similar or improved compared to status on admission for all patients. No adverse events were recorded in any patient before discharge.

Adverse events were graded using CTCAE guidelines. Adverse events (see Supplementary Table 3) were reported in 8 patients and included 45 grade I events, 13 grade II events, and 3 grade III events (Cerebral edema managed medically with steroids occurred during the hospitalization and resolved, and an occipital stroke resolving unrelated to surgical intervention outside the 30-days postoperative period). No grade 4 or 5 events were noted. Patients were assessed for complications until 180 days following surgery. One patient developed a stitch abscess on post-operative day 44 which was treated with simple scalp repair. No patients developed CSF leak at any time.

**Fig. 2:**
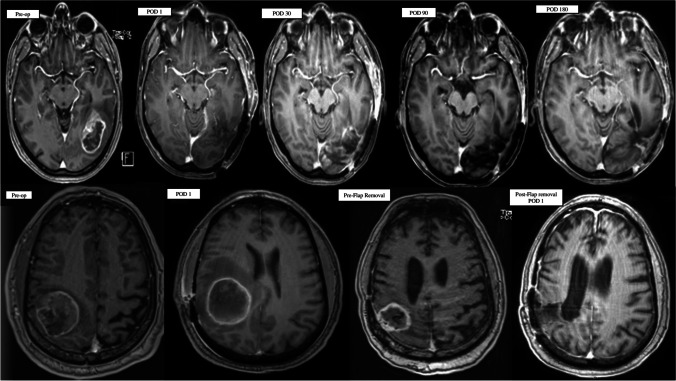
(Upper row) T1 post-contrast MRI of one of the study patients: Pre-operative scan showing an enhancing lesion at the left temporo-occipital region; Post-operative scans at 1, 30, 90, and 180 days after surgery, showing no recurrence or enhancement at the resection site cavity lined by the flap. (Lower Row) T1 post-contrast MRI of one of the study patients with recurrence in the surgical cavity: Preoperative scan showing a right parietal lesion; Post-operative scan showing the flap lining the resection cavity. Pre-Flap Removal scan showing flap enhancement as seen in a follow-up scan done at 25 months. Post-operative scan done on POD 1 after removal of the flap. (MRI: Magnetic Resonance Imaging; POD: Post-operative Day)

Disease progression at the site of the original tumor was identified in only 4 (33%) patients (median 23 months, range 8–25 months). At the time of this manuscript preparation, only 4/12 (33%) of patients have died. Based on the statistical considerations above 9/10 (90%) of our first 10 patients and 10/12 (83.3%) had 6-month PFS. For all 12 subjects, the median PFS was 9.10 months, and the OS was 17.6 months. 4/12 (33%) of patients are still alive for more than two years and our longest surviving patient currently is alive at 60 months. Importantly, in no case was there worsening MRI enhancement within the flap suggestive of tumor infiltration for the duration of the study (see Fig. 2).

In 4/12 cases, the tumor recurred near the initial tumor bed (Fig. 2), with a median time to recurrence of 23 months (range 8–25 months), well beyond the 6 months defined as one of the primary safety points (Supplementary Table 4). In 3/4 of those cases, patients underwent reoperation (biopsy was done in the 1 case). The tumor was re-resected and the flap was removed and sent for histopathological analyses.

**Fig. 3:**
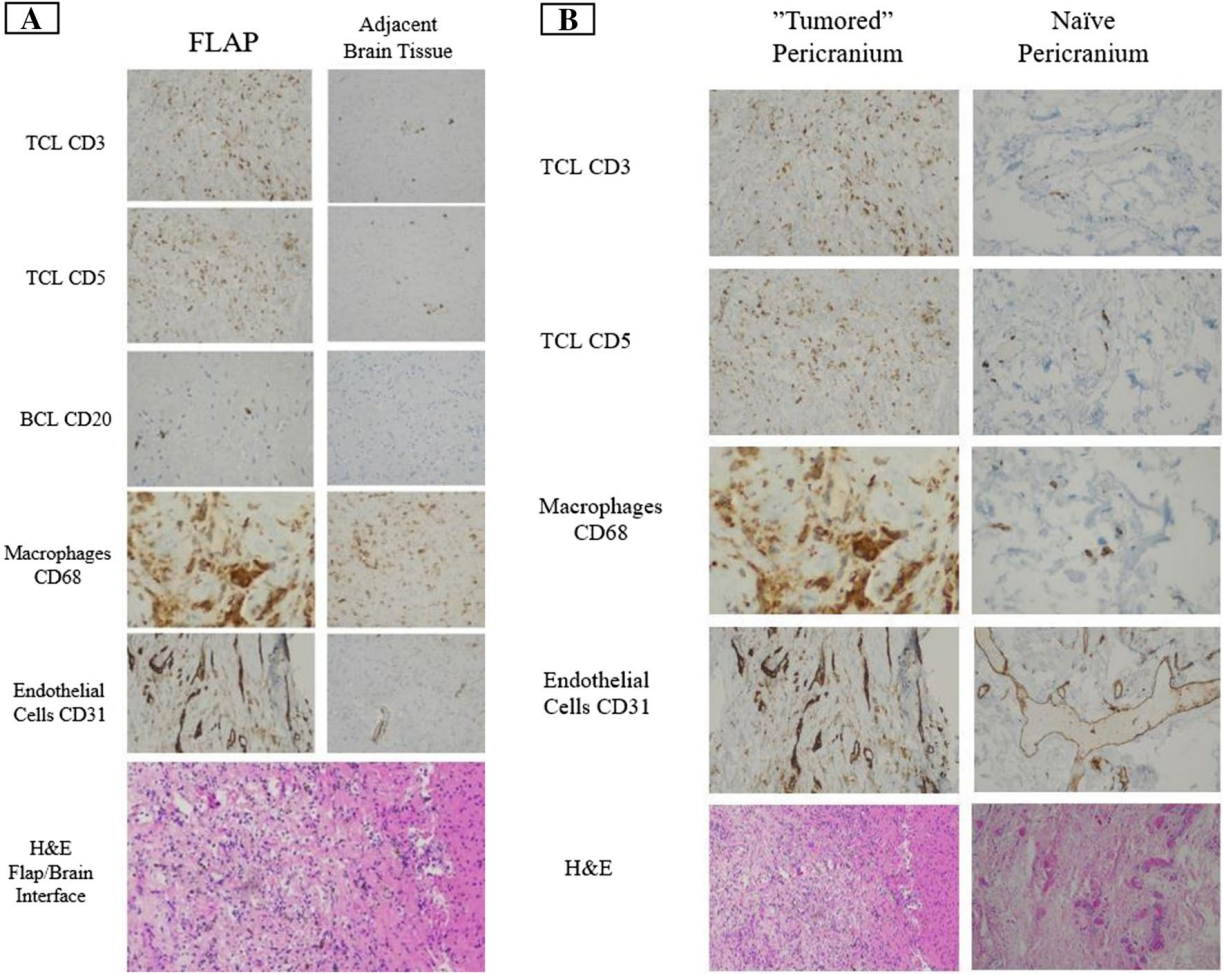
**A** Immunohistochemical and histological slides from a removed flap (Left) compared to adjacent brain tissue (Right), showing the presence of immune system cells within the flap tissue, as well as in the adjacent brain tissue. **B** Immunohistochemical and histological slides from a removed flap compared to naïve pericranial tissue harvested at the time of re-operation, showing the presence of immune system cells and capillaries formed to a greater extent in the peri-cranial "tumored" flap (Left) compared to naïve pericranium (Right)

Interestingly, the removed flap tissue demonstrated significant lymphocytic infiltration. Patient 6 underwent removal of the flap at re-resection and immunohistochemical analysis of the flap (Fig. 3) at 8 months post-insertion. Immunohistochemical analysis of the removed flap demonstrated abundant CD3 and CD5 positive T lymphocytes and some CD20 and PAX 5 positive B lymphocytes. Adjacent brain tissue showed rare CD3 and CD5 positive T lymphocytes but no B lymphocytes supported by negative CD20 and PAX 5 staining. The flap showed abundant small blood vessels which were highlighted by CD31 staining. Notably, the adjacent brain tissue showed rare blood vessels. The flap showed abundant CD68-positive macrophages and the adjacent brain tissue also showed abundant CD68-positive microglia/macrophages.

Control pericranium was sampled at re-resection from an area not implanted into the microenvironment. Immunochemical analysis was compared with analysis of a control naïve peri cranial tissue not implanted into the resection cavity. Histopathological analyses again noted reduced immune cells and fewer capillaries in the naïve pericranium/fascia compared with tissue implanted along the resection cavity suggesting that implantation changed the microcellular composition of the implanted tissue (Fig. 3).

## Discussion

Despite decades of research, the prognosis of GBM remains extremely poor [15]. Apart from the complex and heterogeneous signaling pathways, the unique tumor microenvironment of GBM strengthens the resistance to radiation and chemotherapy [16]. Importantly, the BBB presents a two-fold challenge: First, it prevents many intravenously administered chemotherapeutics from reaching sufficient concentrations in the brain [17]. Second, it limits the immunologic response to tumor-associated antigens in GBM thereby hindering a strong potential immunological response [18]. In this study, we investigated the safety and feasibility of inserting a vascularized temporoparietal fascial or pericranial flap into the tumor cavity as a method of promoting vascular ingrowth devoid of a BBB into the peritumoral microenvironment to facilitate using systemic therapies as well as to facilitate an improved immunologic response against the tumor.

Our results show that TPFF or PCF can be easily harvested, without a significant technical challenge. Furthermore, the pre-defined specific safety goals of this surgical technique were met in this study. Lining the resection cavity with a vascularized TPFF/PCF did not result in increased adverse events following implantation. No patient demonstrated increased seizure activity or wound complications due to devascularization of the overlying scalp. In our statistical analysis, both PFS and OS exceeded 6 months and greater than 70% of patients had 6 m PFS. Most notably, histopathological analyses of resected TPFF/PCF flaps at recurrence demonstrated no tumor infiltration into the flap suggestive of “hijacking” of the flap by the underlying GBM.

Tumors reoccurred at the craniotomy site in 4 patients in our cohort, providing an important opportunity to sample the tissue as it matured within the cavity. In all 4 cases, the presence of lymphocytes within the flap, and to a lesser extent within the adjacent brain, coupled with a lack of neoplastic cells in the transplanted flap, was encouraging as it supports the concept of a vascularized flap as a BBB-circumvented “scaffold”. Similarly, the presence of immune mediators and macrophages supports the introduction of an immunologic, inflammatory response into the tumoral environment [19, 20]. Although in this preliminary analysis, these changes were not identified robustly within the tumor resection bed, this study was not designed to examine this specifically, and future work is necessary to characterize changes in the resection bed related to TPFF/PCF insertion. Future studies using checkpoint inhibitors, for example, may further allow for T-cell infiltration into the tumor microenvironment via the implanted flap.

Previous studies have shown that most recurrences after resection in GBM patients occur in, or adjacent to, the resection cavity [21]. Targeting the cavity, facilitated by a flap, would therefore carry a potential for improved treatment efficacy. Thus, the finding of the increased population of immune cells and capillaries in the flap adjacent to the tumor microenvironment, compared to naïve pericranium, was encouraging [22].

Past studies in both animal models and humans, exploring post-stroke or TBI neovascularization via pial synangiosis in recovering central nervous system tissue, show robust neovascularization [23, 24]. Analyses of these newly formed blood vessels show that the vessels that form either lack or have a substantially more permeable BBB relative to healthy brain tissue [12]. The Matsushima grade, developed to assess these collaterals, describes the extent of perfusion on postoperative angiograms [25, 26]. Interestingly, the angioarchitecture of these vessels bears resemblance to vessels formed after an insult coupled with a microenvironment supporting neuroangiogenesis. knowledge of BBB forming around the bypass collaterals, however, remains limited.

We hypothesized that the insertion of TPFF/PCF vascularized flaps would cause similar vascular ingrowth and potentially facilitate the delivery and effects of local therapy into the perilesional cavity [22]. This would permit access to systemic and targeted therapy which does not normally cross the BBB into the adjacent peritumoral regions which commonly demonstrate the highest potential for tumor recurrence. The implantation of Gliadel wafers at the site of a resected brain tumor could offer sustained high local concentrations of carmustine and has shown effectiveness in a dose-dependent manner [27]. Studies have shown that transposing omentum onto the cerebral cortex can stimulate vessel penetration into the cortex in twelve hours [28]. The potential for omental vessels to form collateral connections with subarachnoid vessels was established in preclinical models of GBM [28]. Another earlier animal study had shown that free muscle grafts can form permeable new vessels on the brain, allowing molecules of different sizes to penetrate, with the depth of penetration correlating to the size of the graft [29]. In our study, the flap showed abundant CD68-positive macrophages and the adjacent brain tissue also showed abundant CD68-positive microglia/ macrophages. Theoretically, we hope that combining our technique with immunomodulatory strategies such as immune checkpoint inhibitors to increase T cell activity, strategies targeting CD8 + T cells, Tregs, and γδ T cells could potentially increase the abundance of immune cells and improve treatment response [30]. More samples are necessary to precisely understand the pathobiology of the interface between the flap and microenvironment of the resection cavity.

While the study met its primary and secondary endpoints, recurrence in a subset of patients permitted histopathological analyses of previously inserted tissue. These analyses identified a source of immune cells in direct contact with the neoplastic tissue. This element can potentially be additive to the hypothesized BBB bypass effect and may generate increased exposure of an immunologically ‘cold’ tumor (GBM) to a naturally lymphocytic-rich implanted tissue [19]. Therefore, if supported by future larger trials, the presence of B-cells, T-cells, and macrophages, as shown in 4 cases presented here both in the flap as well as in adjacent brain tissue, could provide support for a possible local immuno-therapeutic approach.

## Limitations

Our study has several limitations. Due to the nature of a small cohort, some adverse effects which are only clinically noticeable in a larger group, may be missed. The surgical technique described above, although simple to reproduce and not shown to extend the duration of surgery, may not be suitable for fragile patients, or in patients with other systemic diseases that may affect wound healing. Importantly, establishing a clear relationship between the suggested physiological process occurring at the interface of the flap and adjacent brain tissue, could not be demonstrated in this pilot study.

## Conclusions

Our study suggests that pedicled autologous TPF or PCF, lining the resected tumor’s cavity, is both a safe and feasible technique tolerated by newly diagnosed GBM patients. Results show an encouraging outcome in terms of procedure tolerability, safety profile, and disease progression. Further future studies are warranted to confirm the safety and efficacy of this approach in patients with newly diagnosed GBM.

### Supplementary Information

Below is the link to the electronic supplementary material.

Supplementary file1 (DOCX 14 KB)

Supplementary file2 (DOCX 19 KB)

## Data Availability

The datasets generated during and/or analyzed during the current study are available from the corresponding author upon reasonable request.
