# Correction to: Results from a first-in-human phase I safety trial to evaluate the use of a vascularized pericranial/temporoparietal fascial flap to line the resection cavity following resection of newly diagnosed glioblastoma

**DOI:** 10.1007/s11060-024-04701-7

**Published:** 2024-05-17

**Authors:** Omer Doron, Tamika Wong, Faina Ablyazova, Souvik Singha, Julianna Cavallaro, Netanel Ben-Shalom, Randy S. D’Amico, Manju Harshan, Amy McKeown, Avraham Zlochower, David J. Langer, John A. Boockvar

**Affiliations:** 1 Department of Neurosurgery, Lenox Hill Hospital, Donald and Barbara Zucker School of Medicine at Hofstra/Northwell Health, 130 East 77Th Street, 10075 New York, NY USA; 2 Department of Biomedical Engineering, The Aldar and Iby Fleischman Faculty of Engineering, Tel Aviv University, Tel Aviv, Israel; 3 Department of Pathology, Lenox Hill Hospital, Donald and Barbara Zucker School of Medicine at Hofstra/Northwell Health, 130 East 77Th Street, 10075 New York, NY USA; 4 Department of Radiology, Lenox Hill Hospital, Lenox Hill Hospital, Donald and Barbara Zucker School of Medicine at Hofstra/Northwell Health, 130 East 77Th Street, 10075 New York, NY USA

**Correction to: Journal of Neuro-Oncology**

10.1007/s11060-024-04647-w

In this article, Table 2 included the following errors:

Under the heading “Tumor Location”:

Case 4: it should be “Left Temporal” instead of “Right Temporal”

Case 5: it should be “Right Parietal” instead of “Left temporal”

Case 6: it should be “Left Frontal” instead of “Right frontoparietal”

Case 7: it should be “Right Parietal” instead of “Right frontal”

Under the heading “Surgical Approach”:

Case 10: it should be “Left Frontal” instead of “Right Frontal”

The original article has been corrected.

