# Supplementary material for: Results from a first-in-human phase I safety trial to evaluate the use of a vascularized pericranial/temporoparietal fascial flap to line the resection cavity following resection of newly diagnosed glioblastoma
Source: J Neurooncol. 2024 Apr 26;168(2):225–35. doi: 10.1007/s11060-024-04647-w (PMC11147875; doi:10.1007/s11060-024-04647-w)
Supplement: Supplementary file 1 — Supplementary file1 (DOCX 14 KB) [file 11060_2024_4647_MOESM1_ESM.docx]

**Table 3.** Adverse events (No grade 4 or 5 adverse events were noted in this study)

**System Grade 1 Grade 2 Grade 3**

General 4 3 0

Neurologic 21 5 2

Respiratory 0 0 0

Cardiovascular 1 2 0

Gastrointestinal 4 0 0

Genitourinary 7 0 0

Integumentary 1 2 0

Musculoskeletal 1 0 0

Metabolic 0 0 0

hematologic 0 0 1

Psychiatric 4 1 0

ENT 2 0 0
