# Supplementary material for: Results from a first-in-human phase I safety trial to evaluate the use of a vascularized pericranial/temporoparietal fascial flap to line the resection cavity following resection of newly diagnosed glioblastoma
Source: J Neurooncol. 2024 Apr 26;168(2):225–35. doi: 10.1007/s11060-024-04647-w (PMC11147875; doi:10.1007/s11060-024-04647-w)
Supplement: Supplementary file 2 — Supplementary file2 (DOCX 19 KB) [file 11060_2024_4647_MOESM2_ESM.docx]

**Table 4.** Flap characteristics.

| Case No. | Flap type (TPFF/ PCF) | Mass Effect (Y/N) | MRI findings at 6-months post-operatively | Re-operation? (Y/N), Indication | Time to Re-operation (Months) | Flap histology if available |
| --- | --- | --- | --- | --- | --- | --- |
| 1 | TPFF | N | Enhancement | Y, Remote progression | 5 | N/A |
| 2 | TPFF | N | Enhancement | Y, Biopsy | 25 | Fibrous tissue, negative for tumor. |
| 3 | TPFF | N | No enhancement | N | N/A | N/A |
| 4 | TPFF | N | No enhancement | N | N/A | N/A |
| 5 | PCF | N | Enhancement | Y, Flap removal | 23 | Flap with necrosis and calcifications, negative for tumor |
| 6 | TPFF | N | No enhancement | Y, flap removal | 8 | Flap with necrosis, negative for tumor. |
| 7 | PCF | N | No enhancement | N | N/A | N/A |
| 8 | PCF | N | No enhancement | N | N/A | N/A |
| 9 | PCF | N | No Enhancement | N | N/A | N/A |
| 10 | PCF | N | No Enhancement | Y | 8 | Flap with necrosis and calcifications, negative for tumor |
| 11 | PCF | Y | Enhancement | N | N/A | N/A |
| 12 | PCF | N | Enhancement | N | N/A | N/A |

(TPFF: Temporoparietal fascial flaps; PCF: Peri Cranial Flaps Y: Yes; N: No; N/A: Not Available;)
